# Small molecule modulation of microbiota: a systems pharmacology perspective

**DOI:** 10.1186/s12859-022-04941-2

**Published:** 2022-09-29

**Authors:** Qiao Liu, Bohyun Lee, Lei Xie

**Affiliations:** 1 Department of Computer Science, Hunter College, The City University of New York, New York, NY USA; 2 Ph.D. Program in Computer Science, The City University of New York, New York, NY USA; 3 Ph.D. Program in Biochemistry and Biology, The City University of New York, New York, NY USA; 4 Helen and Robert Appel Alzheimer’s Disease Research Institute, Feil Family Brain and Mind Research Institute, Weill Cornell Medicine, Cornell University, New York, NY USA

**Keywords:** Microbe–microbe interaction network, Polypharmacology, Drug discovery, Systematical biology

## Abstract

**Background:**

Microbes are associated with many human diseases and influence drug efficacy. Small-molecule drugs may revolutionize biomedicine by fine-tuning the microbiota on the basis of individual patient microbiome signatures. However, emerging endeavors in small-molecule microbiome drug discovery continue to follow a conventional “one-drug-one-target-one-disease” process. A systematic pharmacology approach that would suppress multiple interacting pathogenic species in the microbiome, could offer an attractive alternative solution.

**Results:**

We construct a disease-centric signed microbe–microbe interaction network using curated microbe metabolite information and their effects on host. We develop a Signed Random Walk with Restart algorithm for the accurate prediction of effect of microbes on human health and diseases. With a survey on the druggable and evolutionary space of microbe proteins, we find that 8–10% of them can be targeted by existing drugs or drug-like chemicals and that 25% of them have homologs to human proteins. We demonstrate that drugs for diabetes can be the lead compounds for development of microbiota-targeted therapeutics. We further show that the potential drug targets that specifically exist in pathogenic microbes are periplasmic and cellular outer membrane proteins.

**Conclusion:**

The systematic studies of the polypharmacological landscape of the microbiome network may open a new avenue for the small-molecule drug discovery of the microbiome. We believe that the application of systematic method on the polypharmacological investigation could lead to the discovery of novel drug therapies.

**Supplementary Information:**

The online version contains supplementary material available at 10.1186/s12859-022-04941-2.

## Background

As the most abundant organism, symbiotic microbiome biomasses in human body sites are as rich as the human somatic cells [1]. Traditional culture-based or non-culture-based methods only detect limited groups of microbes, restricting our scopes on a comprehensive understanding of the entire microbial community. Advances in high throughput sequencing technology substantially enhance our powers to characterize the microbial community. Up to date, thousands of microbe genomes have been sequenced [2]. These large scale sequencing data collected have driven forward a myriad of intriguing researches, including finding microbiome biomarkers [3, 4], investigating their association with diseases [5, 6], and uncovering the dynamicity of microbial community [7, 8].

Small-molecule drugs offer new opportunities and has emerged as a new frontier for microbiome drug discovery and precision medicine [9]. It has been shown that small molecule drugs, like antibiotics, relieve bacterial infection symptoms by controlling the overgrowth of pathogens [10]. However, many microbe species have developed drug resistance mechanism, especially to several widely used antibiotics [11, 12]. This raises the requirement for the investigation and discovery of new drugs. The second concern that comes with drug intervention treatment is their adverse effect on other microbes, non-pathogenic species or even probiotics [13, 14]. Drug intervention causes microbiota compositional change. The current view believes that microbiota homeostasis is a crucial healthy feature of our "forgotten organ" [15]. Elimination or diminution of healthy commensal microbes draws dysbiosis in our body site ecologically, and pathogenic microbes take this advantage to causes symptoms like diarrhea and nausea [13]. Besides, the dysbiosis is found to associated with many diseases, including obesity [16], allergy [17], type 1 diabetes (T1D) [18] and type 2 diabetes(T2D) [19], inflammatory bowel disease (IBD) [20], rheumatoid arthritis (RA) [21], autism [22] and cancer [23]. For instance, T1D studies have shown that the abundance of Bacteroides in patient group is higher than that in the control group [18]. Thus drugs minimizing side effects on other symbiotic microbes are desired.

Emerging endeavors in small-molecule microbiome drug discovery continue to follow a conventional “one-drug-one-target-one-disease” process. It is often insufficient and less successful in tackling complex systematic diseases. Systems pharmacology, which aims to modulate multiple microbe targets in the microbiome-microbiome interaction network, could be a potentially powerful approach for microbiome drug discovery that can solve the aforementioned concerns. There are many data-driven methods and text-mining-based approaches to construct a microbe–microbe network based on existing evidence. For data-driven methods, the networks were inferred based on microbiome co-occurrence in the host [24, 25] or dynamic changes in a time course [26, 27]. However, these methods still suffer many issues, such as the challenge to get comparable absolute microbe abundance [28, 29], the difficulty in biological interpretation and the requirement of optimization of sampling strategies [30, 31]. Another popular approach for constructing the microbe–microbe association network is through automatic text-mining [32, 33]. These methods are error-prone, biased, and lack biological representation of complexed microbiome-microbiome associations such as competition and corporation.

Microbiome network can also be reconstructed by knowledge-driven approaches. Within these methods, network reconstruction is based on knowledge of curated metabolites and biochemical reactions. In one study, a global interspecies metabolite interaction network, NJS16, is constructed using microbe metabolites consumption and production information and it elucidates the interplay between different species in human gut [34]. This network is used as the foundation for building a context-specific network, MIN, which models growth rate effect by other species or microbiome community under certain conditions [34, 35]. NJS16 has also been applied in the construction of a multi-level trophic model of gut microbiome, which is used to simulate the metabolites flows across microbes [36].

Here we constructed a disease-centric gut microbial community network to model microbes and microbial community effects on host health. By inferring the microbe–microbe relationship from their metabolites input and output profiles collected from aforementioned genome-scale metabolic modeling [34], we simulate the propagating process of microbe effect on host health using a new Signed Random Walk with Restart algorithm (SRWR) [37]. We annotated 104 microbe nodes in the network based on their effects on host health by manually literature review. To our knowledge, it is the first time to integrate the effect of the microbiome on host health into a microbiome-microbiome interaction network model. Additionally, our network represents mechanistic relationships between microbiomes and encodes them by positive or negative signed edges. By contrast, all existing networks have only a single positive-signed edge and cannot model complex interactions between microbiomes. This unique node-labeled signed network model enabled us to predict the physiological roles of 409 unannotated microbes in the network as potential pathogenic or commensal using SRWR [37]. It is noted that conventional Random Walk with Restart (RWR) and other state-of-the-art methods such as Graph Neural Network [38–40] only work on networks with positive-weighted edges. Because our network has also negative-weighted edges, a method that can model the signed network such as SRWR is needed. Moreover, we need to predict complex label propagations such as friend-of-enemy or enemy-of-enemy to determine if an unannotated microbiome is pathogenic or commensal. SRWR is designed to support this type of analysis, and more powerful than Signed spectral Ranking (SR) [41] and Modified PageRank (MPR) [42].

In order to realize systems pharmacology of microbiome, many unanswered questions remain: what the chemical space is in which the chemical compounds will inhibit pathogenic microbiome interactions but not disturb commensal microbiome? if we can target multiple pathogenic microbiomes, at the same time not inhibit commensal microbiomes? To address these questions, we performed a survey on current knowledge of drugs-targets interactions and found a significant number of genes that could potentially be drug targets in each microbe based on sequence homology. Our analyses suggested that a large number of genes have homologs to existing drug targets. We also identified a list of potential protein targets specific to the pathogenic microbe and not to the commensal microbe. Our analyses considered how microbiome interplays with each other at the metabolites level and how drugs affect microbe growth through genome analysis. This application is not limited to the exemplar analysis performed here. The systematic studies of polypharmacological landscape of microbiome network may open a new avenue for the small molecule drug discovery of microbiome.

## Results

### A novel disease-centric microbe–microbe interaction network

**Fig. 1:**
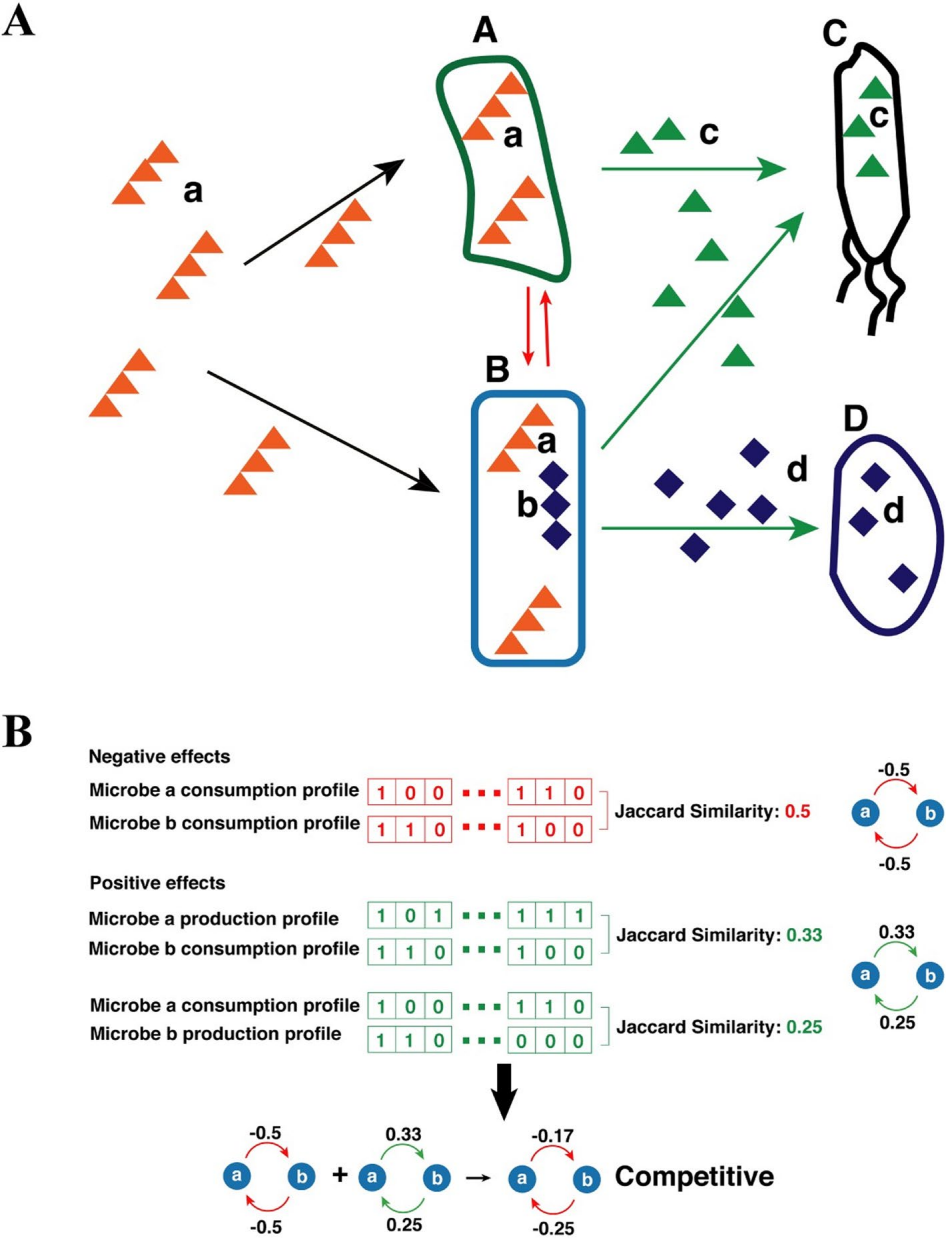
Illustration of relationships between microbiomes. (**A)** Microbe A and B compete for metabolite a and has a negative effect on each other. This negative relationship is shown with a red arrow. Microbe A and microbe C have a cross-feeding relationship. Microbe A can degrade macromolecule a into metabolite **c**, which can be taken by microbe **C**. Cross-feedings also exist between microbe B and C, and between microbe B and D through metabolite c and d, respectively. (**B**) An example for the calculation of the relationship between two microbes. Negative effects are calculated as the Jaccard similarity between microbe’s consumption profiles. The positive effect is calculated as the Jaccard similarity of one microbe's consumption profile and another's production profile. The final effect is the aggregation of the negative effect and the positive effect

**Fig. 2:**
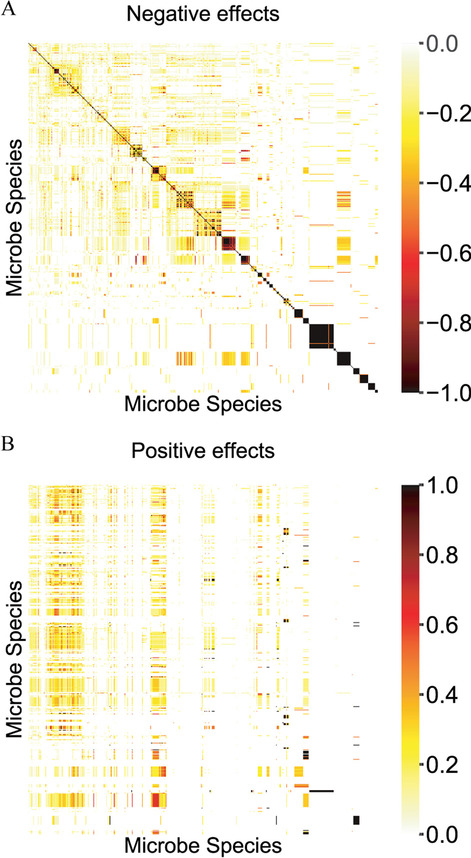
The Jaccard similarity among microbes' metabolite consumption and production profiles. **A** microbe–microbe negative effect score matrix computed as the Jaccard similarity of their metabolite consumption profiles. **B** microbe–microbe positive effect score matrix computed as the Jaccard similarity of one microbiome's metabolite consumption profile and another’s metabolite production profile

We here proposed a new microbe–microbe interaction network, which is inferred from each microbe's metabolite consumption and production profile. Microbes affect each other through different mechanisms. (1) They have negative effects on each other through competing for the same metabolite resources (Fig. 1A). (2) One microbe can have positive effects on others through cross-feeding (Fig. 1A). (3) They can affect other microbes positively or negatively by alternating their living environment, like the change of pH. (4) They could also form predator–prey relationships. We characterize the first two relationships between microbes through inferring an interaction network using microbes' metabolite consumption and production profiles (Fig. 1B). To be specific, the extent of negative relationship is calculated as the Jaccard similarity of two microbe’s metabolite consumption profiles (Fig. 2A). Intuitively, the more metabolites two microbes consume in common, the higher the negative effect they have on each other. On the other side, the positive effect is due to the cross-feeding relationship. The extent of positive effect is calculated as the Jaccard similarity of one microbe's production profile and the other's consumption profile (Fig. 2B). It is worth mentioning that the positive relationship between the two microbes is not symmetrical. Finally, the extents of positive effect and negative effect are summarized to generate the final edge weights.

**Fig. 3:**
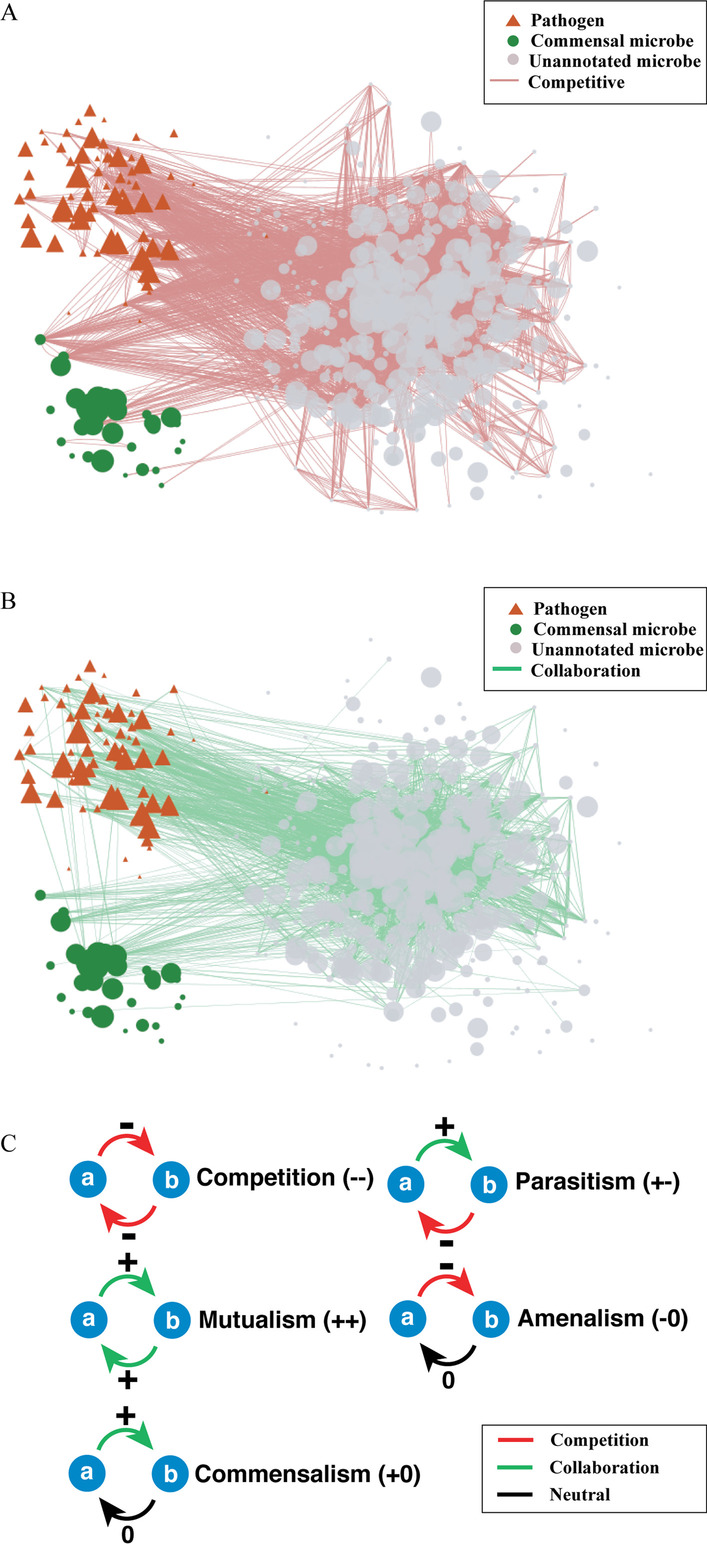
Illustration of Microbe–microbe network. **A** Negative relationships (red edges) between microbes. Only the edges with weight lower than − 0.7 are shown for simplicity. **B** Positive relationships (green edges) between microbes. Only the edges with a weight higher than 0.5 are shown for simplicity. **C** 5 relationships between microbes, competition (–), mutualism (+ +), commensalism(+ 0), parasitism (+ −) and amenalism(− 0) are shown. −, + and 0 denotes a negative effect, a positive effect, and no effect, respectively. pathogenic and commensal microbes curated through literature review are labeled in red and green, respectively. Other microbes are labeled in gray

The microbe's metabolite consumption and production profiles were curated elsewhere [34]. We include 513 microbe nodes in our network analysis (Fig. 3A and B). These 513 nodes form strongly connected component in the network. This network has the following merits: (1) The graph can thoroughly represent various types of microbe–microbe relationships because the graph is directed. The relationships are not limited to competition (++) and mutualism (−−), where microbes can positively affect each other and negatively affect each other in both directions (Fig. 3C). “ + ” or “−” indicates that the microbe has a positive or negative effect on the other in one direction, respectively. It can also represent more diverse relationships, including the commensalism (+ 0), parasitism (+ −) and amenalism (− 0). 0 here indicates that no relationship is found in a specific direction. (2) It is biologically meaningful and straightforward to interpret. (3) This microbe–microbe interaction network can avoid the problem in the construction of microbiome network based on the abundance correlation of microbiomes, such that the correlation is sensitive to the data compositionality and is affected by low-abundance [43, 44]. (4) It can be integrated with additional networks that are derived from other information (e.g., environmental factors) into a more sophisticated heterogeneous network analysis framework.

As the "forgotten organ" of the human body, the microbiome can help to digest dietary macromolecules in gut [45], protect against many common infections [46, 47] and maintain human health. On the other hand, infection of pathogenic species can cause severe disorders or diseases. The diverse effects of microbes on human health make it challenging to analyze and predict the impact of the whole microbiome ecosystem on human health. Many studies have shown that network analysis can effectively model the relationship and interplay between different species in microbiome [48]. These interactions are usually represented by the edges connecting two microbe nodes. A weight is assigned to denote the association extent and type. However, to our knowledge, these networks haven’t included information about the effect of microbes on health. The network that combines the information of both microbe–microbe interaction and microbe effect on health should be used for analyzing the influence of microbiome ecosystem on host health. We manually curated the microbe function on host health through the literature review. Out of 513 microbes in our network, we curated 72 microbes having pathogenic effects, either as a cause of disease or a contributor of human illness, and 32 microbes having commensal effects, helping macro-molecules digestion or preventing from infections (Additional files 2 and 3). Majority of microbes are still understudied, so their influences on human health are not determined due to either lack of evidence or ambiguous descriptions. Some species have different strains which have different influence on human health [49]. The manually curated information about microbe effects on human health is then added in the network as the microbe node attributes. The commensal microbe nodes have node weighted as " + 1", while the pathogenic microbe nodes have node weighted as "-1". Other nodes that are left unlabeled are then annotated with a predicted value using the known microbe nodes attribute and microbe–microbe interaction edge information with a Signed Random Walk with Restart algorithm.

### Microbe effect annotation with Signed Random Walk with Restart

Because many microbe effects on human health are unknown, we developed a graph mining strategy to infer their effects based on annotated network. Using a Signed Random Walk with Restart (SRWR) model, each unannotated microbial species was treated as a node with an unknown health effect attribute, and tested for how it was influenced by the neighboring nodes in the network through corporation (positive signed edge) and competition (negative signed edge). The advantage of incorporating SRWR model into our analysis was on the fact that the network recognizes both cooperative relationships as well as the competitive relationships, which resembles the true nature of the microbial ecology. The premise of our analysis is that “friend” of “friend” or “enemy” of “enemy” will be “friend”, and “friend” of “enemy” or “enemy” of “friend” will be “enemy”.

To assess the accuracy of our predictions, we use the curated data set of the 32 commensal and 72 potential pathogenic bacterial species that affects human health as a benchmark. Using leave-one-out cross-validation, we obtained confusion matrix with an average F1 score of 0.905. Specifically, the prediction of positive nodes yielded the precision of 0.780 and the recall of 1.000, while the prediction of negative nodes yielded the precision of 1.000 and the recall of 0.875. This accurate SRWR algorithm yielded the prediction of 135 positive nodes (potentially commensal) and 274 negative nodes (potentially pathogenic) for 409 total species without annotations associated with human health (Additional file 4).

### Survey on microbe protein druggability and structural predictability

**Fig. 4:**
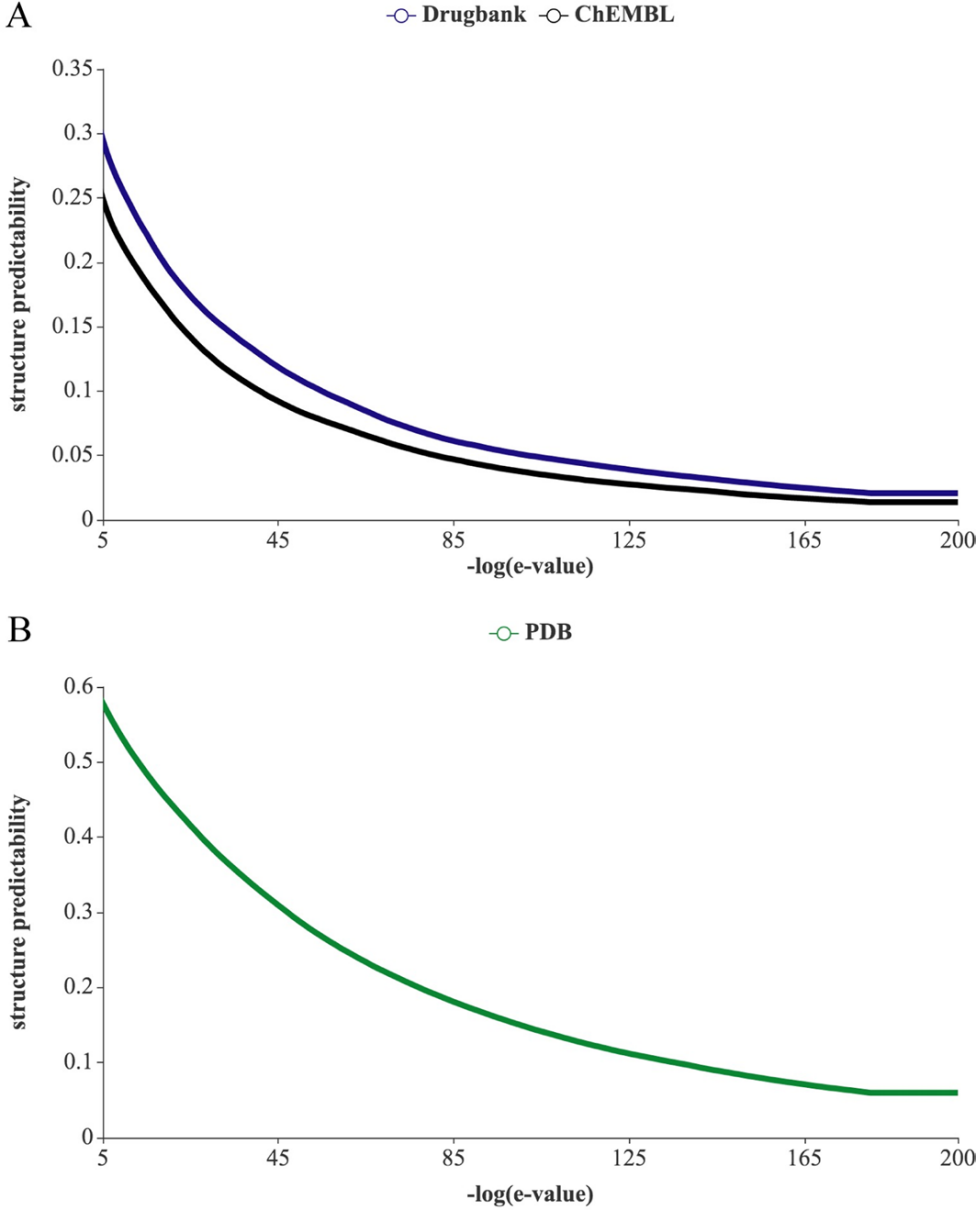
Percentage of protein targets in microbiomes that have sequence homologs in different databases, **A** ChEMBL and Drugbank, and **B** PDB. E-value is the criterion used in the sequence similarity search by BLAST. The lower e-value is, the closer homolog is

A microbe protein is denoted druggable if drugs or drug-like chemicals can target this protein or its homologs (i.e. proteins in the same gene family) [50]. To have a comprehensive view of drug targets space of the microbiome, we included all protein sequences of microbe species collected by the Human Microbiome Project (HMP) in our study [2, 51]. Drugbank and ChEMBL databases are two of the most popular and updated drug-target interaction databases [52, 53]. Up to date, DrugBank and ChEMBL possessed more than 5000 and 15,500 protein target sequences and drug information interacting with these targets. We screened for the homologs of all protein sequences of each microbe species in the target sequences set of each drug-target interaction database using PSI-Blast[54–56]. The e-value resulting from a specific sequence search indicates the number of hits we can get by chance when we search a protein sequence against a database. From the plot of the percentage of protein with homologs in each microbe versus −log (e-value), we determined that the elbow point of curve is at which e-value is around 10e−60 (Fig. 4A). With this e-value as the threshold, we determined that 10% and 8% of microbe protein sequences were found to have close homologs in DrugBank and ChEMBL targets database. Besides, the structure information of protein is critical for the structure-based drug design and polypharmacology [57]. The protein structures saved in Protein Data Bank archive (PDB) are widely used for protein structure prediction, so we searches for the homologs, which show high sequence similarities with microbe proteins, in PDB [58]. With e-value at 10e-60, we show that 25% of microbe proteins have close homologs in PDB (Fig. 4B).

### Drug repurposing analysis shows that drugs for Diabetes have potential to regulate microbiomes

A successful treatment for human diseases caused by microbe infection is antibiotic intervention, which is used to control the overgrowth of one or a group of pathogenic microbes. Due to the overuse of them, many microbes have shown antibiotic resistance [11]. Another problem with some existing drugs is side effects on other symbiotic microbe species, which causes microbiome dysbiosis. Thus, disrupting pathogen interaction network by targeting multiple pathogenic microbiomes but not disturbing commensal microbiomes will be a potential powerful strategy for microbiome drug discovery. Because drug repurposing exhibits more advantages than developing a novel drug [59], we perform a computational screen on FDA approved or investigational drugs for innovative potential drugs for targeting microbes. To avoid undesirable side effects, the drugs should not affect commensal microbes proteins. With this intuition, we search for drugs that can potentially affect simultaneously multiple pathogenic microbes and avoid undesirable effect on commensal microbes.

We performed the screening on two databases: Drugbank and STITCH. Most chemicals in the Drugbank database are drugs that are FDA approved or under investigation, and most of the drug-target interactions have experimental evidence. We collected the drugs that could target proteins that are homologs of proteins in 72 pathogenic microbes and then excluded those targeting on homologs of proteins in 32 commensal microbes. Ultimately, we found 589 drugs that satisfy this constraint (Additional file 5). On the other side, parts of compounds in STITCH are predicted drugs that lack experimental support. STITCH database also possesses predicted drug-targets interaction for each microbe species. Thus the screen includes both drugs and some non-drug compounds. Drug-target interactions in the STITCH database have various types, like inhibition, activation, and catalysis. We conducted more specific screening by considering each interaction type, as described in Methods. On average, one third of compounds in the STITCH database are found to have pharmaceutical usage. Finally, we found 170 drugs that appear in both STITCH screen and DrugBank screen results (Additional file 6).

We then performed drugs overrepresentation analysis of these 170 drugs. The background drug list used in this analysis includes all drugs targeting microbe proteins homologs. Two analyses are conducted with two different drug classification systems, including the anatomical therapeutic chemical classification system (ATC) and the Drugbank classification system. Surprisingly, both analyses demonstrate that the drugs used in Diabetes are the statistically significantly overrepresented drugs categories (Tables 1 and 2). Our predictions are supported by findings of which several anti-diabetic drugs can affect microbiome compositions and may harbors anti-bacterial properties, such as Thiazolidinediones (TZDs) and metformin [60]. Even though no direct study and evidence shows that the sulfonylureas inhibit pathogens, but one study shows indirect evidences of sulfonylureas affecting the microbes involved in gut metabolism [61]. Besides, it is also worth noting that the nitric oxide synthases antagonists & inhibitors are also enriched [62, 63].

**Table 1:** Hypergeometric test on drugs based on drug category information in Drugbank

Categories	*p* Value	Bonferroni corrected *p* value	B&H FDR adjusted *p* value	B&Y FDR adjusted *p* value
Nitric oxide synthase antagonists & inhibitors	0.00000	0.00012	0.00012	0.00082
Urea	0.00000	0.00029	0.00014	0.00094
Sulfonylureas	0.00004	0.01525	0.00508	0.03327
Oral hypoglycemics	0.00007	0.02843	0.00711	0.04652
Drugs used in diabetes	0.00018	0.06979	0.01204	0.07882
Sulfonylurea compounds	0.00019	0.07226	0.01204	0.07882
Stereoisomerism	0.00023	0.08839	0.01263	0.08264
Aldehyde reductase, antagonists & inhibitors	0.00041	0.16059	0.02007	0.13138
Enzyme inhibitors	0.00047	0.18206	0.02023	0.13239
Blood glucose lowering agents	0.00061	0.23983	0.02398	0.15696
Pyrazoles	0.00135	0.52458	0.04769	0.31211

Only the categories with Bonferroni corrected *p* value, B&H FDR adjusted *p* value, or B&Y FDR adjusted p-value lower than 0.05 are shown

**Table 2:** Hypergeometric test on ATC code information

Levels	*p* value	Bonferroni	B&H FDR adjusted *p* value	B&Y FDR adjusted *p* value	Description
A10BB	0.00002	0.00088	0.00088	0.00407	Sulfonylureas
A10B	0.00005	0.00298	0.00149	0.00689	Blood glucose lowering drugs, excl. insulins
A10	0.00013	0.00768	0.00256	0.01185	Drugs used in diabetes
A10BX	0.00087	0.04934	0.01234	0.05710	other blood glucose lowering drugs, excl. insulins
A	0.00158	0.09024	0.01805	0.08355	Alimentary tract and metabolism

Only the categories with Bonferroni corrected p-value, B&H FDR adjusted *p* value, or B&Y FDR adjusted *p* value lower than 0.05 are shown

### Characterization of potential targets specific in pathogenic microbe proteins

We then identify targets that are homologs of pathogenic microbes' proteins but not those of commensal microbes' proteins. The results can assist in discerning the potential directions in drug discovery. The scope of target identification is limited to the protein targets collected in the Drugbank database. We selected 462 potential proteins. (Additional file 7). Functional enrichment analysis was then performed on these selected targets with DAVID [64, 65]. The background targets include all found homolog targets of microbes' proteins that are collected by sequence search against the Drugbank database. The results show that proteins in periplasmic and cellular outer membrane are overrepresented (Table 3). The statistically significant enriched functional annotations are signal proteins and transport proteins.

**Table 3:** Results from protein targets functional annotation analysis with DAVID

Categories	Bonferroni	B&H FDR adjusted *p* value	B&Y FDR adjusted *p* value
Periplasm	0.00001	0.00001	0.00007
Signal peptide	0.00010	0.00010	0.00022
Topological domain: periplasmic	0.00045	0.00022	0.00100
GO:0009279 ~ Cell outer membrane	0.00011	0.00011	0.00104
Cell outer membrane	0.00028	0.00014	0.00133
GO:0030288 ~ Outer membrane-bounded periplasmic space	0.00097	0.00048	0.00888
Transmembrane beta strand	0.00281	0.00094	0.01348
Signal	0.00555	0.00139	0.02669
Transport	0.01127	0.00226	0.05434

Only the categories with Bonferroni corrected *p* value, B&H FDR adjusted *p* value, or B&H FDR adjusted *p* value lower than 0.05 are shown

## Discussion

Existing small-molecule microbiome drug discovery follows conventional one-drug-one-gene-one-species paradigm [9], and focuses on preventing infection or fighting against one microbe, barely considering the microbiome as an ecosystem. We believe systems pharmacology approaches are necessary to identify small molecule drugs for modulating the microbiome ecosystem. With the awareness of the complexity and diversity of microbiota, the reconstruction of microbiota networks is a critical step to study the microbial community and to realize systems pharmacology, and it draws increasing interests [66]. Besides occurrence abundance correlation-based methods, exploring microbes growth sources and chemical products is crucial to elucidate the mechanism of interplay between microbes.

Our disease-centric microbe–microbe network, constructed based on literature review and computational prediction, is still expected to improve and grow over time. Currently, the label of each node is based on species level. This method introduces ambiguity when defining each species effect on health. For instance, *E. coli*, possess harmless and commensal strains in the human gut, in the meantime, some strains are pathogenic and even carcinogenic [49]. However, we believe that our network reflects the general effects of microbiota on health, and are useful. Most of the abundant microbes, which we include in our network, have been well studied regarding their metabolites and effects on human health. Other microbes’ effect on health inferred with the SRWR method covered the information about how they affect human health by interplaying with abundant microbes.

To incorporate host information and environmental factors into the construction of a heterogeneous microbe–microbe interaction network can further enhance our understanding of the microbial community. Previous studies showed that environmental factors are another crucial factors that determine the diversity and composition of the microbial community [67]. Gut microbiota, as the most abundant microbial community, can be affected by personal daily diet and lifestyle [68]. For example, loss of sleep could increase the ratio of Firmicutes to Bacteroidetes [69]. Microbial community is believed to harbor discrete homeostasis states and transit between different states when experiencing environmental changes, at least for skin or vaginal microbiota [70, 71]. Thus, constructing a heterogeneous human–environment–microbiome network will be an important direction for the future work.

The contemporary medical system undergoes an era of transition from traditional population based diagnosis and treatment to a more precise personalized medicine. Microbiota demonstrates high variability via developing different biogeographic signatures of human body sites [72, 73]. Small molecular drug discovery based on patient particular microbiome signatures improves and assists in generating more efficient personalized diagnosis and treatment to cure disease. Our work provides the prime landscape of small molecule drug discovery by exploring the connection between microbe's genome and potential drugs.

## Conclusion

In this paper, we systematically investigated the polypharmacological landscape of the microbiome network. We found that a large number of proteins in pathogen microbes are potential drug targets and inhibiting them may not significantly affect the human host. We further showed that the potential drug targets that specifically exist in pathogenic microbes are periplasmic and cellular outer membrane proteins. We proposed drugs for diabetes can be the lead compounds for development of microbiota-targeted therapeutics. This study may open a new avenue for the small-molecule drug discovery of microbiome for novel drug therapies.

## Methods

### Microbiome interaction network

Microbe species metabolite consumption and production information were manually curated elsewhere [34]. 513 microbe species are included in this dataset. Distribution of the number of metabolites each microbe consumes or produces, and distribution of the number of microbes each metabolite associates with are investigated (Additional file 1). We hypothesize that the final relationship between the two microbes is composed of a negative relationship (competition) and a positive relationship (corporation). The negative extent, negative_ab_, is calculated as the Jaccard similarity of metabolite consumption profile between microbe a and microbe b.

$${Negative}_{ab}= \frac{|{C}_{a} \cap {C}_{b}|}{\left|{C}_{a}\right|+\left|{C}_{b}\right|-|{C}_{a} \cap {C}_{b}|}$$

${C}_{a}$ and ${C}_{b}$ are the consumption profile of microbe a and microbe b. 233 metabolites are investigated and are consumed by at least one microbe species. The positive extent, positive_ab_, is calculated as the Jaccard similarity of microbiome a's metabolites consumption profile to microbiome b's production profile.

$${Positive}_{ab}= \frac{|{C}_{a} \cap {P}_{b}|}{\left|{C}_{a}\right|+\left|{P}_{b}\right|-|{C}_{a} \cap {P}_{b}|}$$

${C}_{a}$ and ${P}_{b}$ are the consumption profile of microbe a and production profile of microbe b. The final microbiome interaction network is a directed graph.

### Signed Random Walk with Restart (SRWR)

The dynamicity of microbiome interaction network with selected 513 microbial species is simulated using Signed Random Walk with Restart algorithm [37]. To predict the label (pathogenic or commensal) of a unannotated microbe species, it is initialized as a start node. For each run, initial score of 1.0 is assigned to the start node with an unknown sign, and then this score is distributed out to the neighboring nodes via edges in the network as the walk goes with random probability. Positive edge would increase the positive ranking score of the neighboring node with the balance attenuation probability of β = 0.5, and the negative edge would increase the negative ranking score of the neighboring node with the probability of γ = 0.5. When the walk is complete, positive scores from known commensal species, and negative scores from known pathogenic species are summed up to predict the label for the unknown start node (Additional files 2, 3, and 4).

### Microbe proteins druggability survey

Protein sequences of microbes are downloaded from Human Microbiome Projects (HMP) [2, 51]. Druggable target sequences are downloaded from Drugbank (www.drugbank.ca) and ChEMBL websites (www.ebi.ac.uk/chembl) [52, 53]. They are saved as fasta format and reformatted to be a Blast database using PSI-Blast tools [54–56]. Microbe protein sequences are searched against each target sequence database to find their homologs. Biopython package is used to perform sequence comparison using PSI-Blast [54–56]. All sequence search results with e-value lower than 10e-4 are saved for further analysis. Scripts used for analysis are available in https://github.com/qiaoliuhub/drug_target_analysis_on_microbiome.

### Potential drugs screening

#### Drugbank

The drug-target interaction database is downloaded from Drugbank (www.drugbank.ca) [52]. By using the homolog targets from the sequence search, we collect the drugs that potentially target microbe proteins for each microbe. All drugs that potentially target pathogenic microbes are gathered into a candidate list, then parts of the drugs in the list are excluded if they can potentially target commensal microbes. 589 drugs in the candidate list are left after screening.

#### STITCH

STITCH database is downloaded from http://stitch.embl.de [74]. STITCH database has grouped drug-target interactions based on microbe species. These drug-target interactions are also classified into different types, such as inhibition, activation, or catalysis. We focus on two interaction types: inhibition and activation. Our primary purpose is to screen for FDA approved or investigational drugs, so we excluded non-drugs compounds. We utilize the STITCH drug ID information, which is also the same with PubChem compound ID, to retrieve the pharmaceutical function information from the PubChem database by using PUG REST API and E-utilities tools [75]. We perform the following screen: (1) Compounds that activate targets in pathogenic microbes but not activate targets in commensal microbes (134 drugs). (2) Compounds that activate targets in pathogenic microbes but not inhibit targets in commensal microbes (431 drugs). (3) Compounds that inhibit targets in pathogenic microbes but not inhibit targets in commensal microbes (185 drugs). (4) Compounds that inhibit targets in pathogenic microbes but not activate targets in commensal microbes (1325 drugs) (Additional file 8).

#### The intersection of Drugbank screening and STITCH screening result

The InChIKey information of all drugs found in Drugbank screening is retrieved from Drugbank full database XML file. The InChIKey information of all drugs found in STITCH screening is collected from the Pubchem website using PUG REST API and E-utilities tools. The intersection of these two drugs InChIKey set is found for later analysis.

### Overrepresentation analysis

#### Drug overrepresentation analysis

All selected drugs’ ATC code and Drugbank classification information are accumulated from Drugbank full database XML file. These two classification systems have hierarchical structures, and all categories in all levels are included. A hypergeometric test is performed on the Drugbank screened 589 drugs list. The Bonferroni correction, Benjamini & Hochberg'sHochberg's FDR adjustment, and Benjamini & Yekutieli'sYekutieli's FDR adjustment methods are used to adjust the p-values of these multiple comparisons. ~ 3700 drugs, which are found to target at least one microbe protein homolog, are used as background drugs list (Additional file 9).

#### Protein targets functional enrichment analysis

462 potential protein targets are filtered out using the Drugbank target sequences database and saved with their UniProt accession numbers. Potential targets functional enrichment analysis is conducted with the database for annotation, visualization, and integrated discovery (DAVID) [64, 65]. The list of 462 potential targets' UniProt accession numbers was uploaded to DAVID as a test gene set. Background gene set includes ~ 1700 microbe protein homologs found in Drugbank (Additional file 10).

### Term definition

- **Microbiome**: Collection of all microbes (bacteria, fungi, and viruses) that are naturally live in the human body.
- **Pathogen microbe**: A microbe that causes diseases.
- **Commensal microbe**: A microbe that has a neural relationship (neither benefit nor harm) with the host.
- **Competition**: Microbes compete with each other for survival.
- **Mutualism**: Microbes are mutually dependent.
- **Commensalism**: Microbes neither benefit nor harm each other.
- **Parasitism**: Microbes live on other microbes.
- **Amenalism**: A microbe inhibits another microbe, but itself is not affected.
- **Systems pharmacology**: A drug discovery paradigm that aims to modulate multiple microbe targets in the microbiome-microbiome interaction network.
- **Polypharmacology**: A compound can inhibit or activate multiple targets simultaneously.
- **Drug repurposing**: Use of existing drugs for different clinical indications from the original one.

## Supplementary Information

**Additional file 1**. **Figure S1**. (A) Distribution of number of metabolites each microbiome consume or produce. (B) Distribution of number of microbiomes each metabolite.

**Additional file 2. Table S1**. Microbes with pathogenic effects on human health by manually literature review.

**Additional file 3. Table S2**. Microbes with commensal effects on human health by manually literature review.

**Additional file 4. Table S3**. The Microbes with unknown effects in literature reviews and their SRWR inferred microbe effects.

**Additional file 5. Table S4**. Drug screen results using Drugbank database.

**Additional file 6. Table S5**. Drugs that are found in both drug screen results using Drugbank database and that using STITCH database.

**Additional file 7. Table S6**. Potential homolog proteins that have homologs with pathogenic microbe proteins but do not have homologs with commensal microbe proteins.

**Additional file 8. Table S7**. Drug screen results in STITCH database.

**Additional file 9. Table S8**. Background drugs list used in drug overrepresentation analysis.

**Additional file 10. Table S9**. Background targets list used in target functional annotation analysis.

## Data Availability

The datasets generated and/or analyzed during the current study are either available in the repository, https://github.com/qiaoliuhub/drug_target_analysis_on_microbiome or included in this published article and its additional files.

## Abbreviations

T1D: Type 1 diabetes
T2D: Type 2 diabetes
IBD: Inflammatory bowel disease
RA: Rheumatoid arthritis
SRWR: Signed Random Walk with Restart
SR: Spectral ranking
MPR: Modified PageRank
