# Supplementary figures and images for: Small molecule modulation of microbiota: a systems pharmacology perspective
Source: BMC Bioinformatics. 2022 Sep 29;23(Suppl 3):403. doi: 10.1186/s12859-022-04941-2 (PMC9523894; doi:10.1186/s12859-022-04941-2)

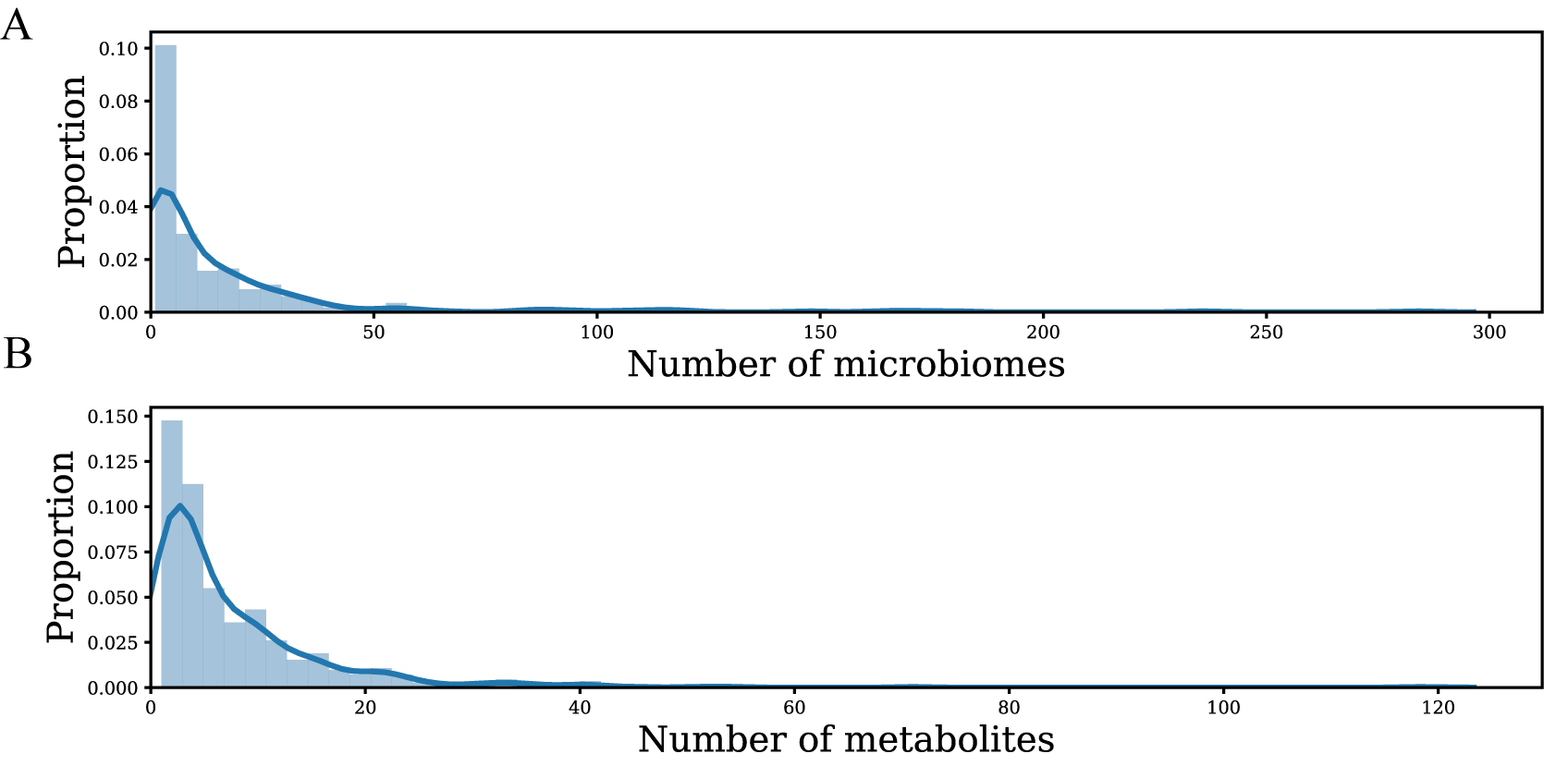

Supplement: Supplementary file 1 — Additional file 1. Figure S1. (A) Distribution of number of metabolites each microbiome consume or produce. (B) Distribution of number of microbiomes each metabolite. [file 12859_2022_4941_MOESM1_ESM.tif]
